# 

**Published:** 1889-02

**Authors:** 


COMMUNICATIONS.

Dental Transplantation and Replantation.... 57
Shedding of Temporary Teeth as to the Process.... 65
The Physician and Dentist.... 69
MONTHLY DIGEST.... 75 and 84

PROCEEDINGS.

Odontological Society of Pennsylvania.... 100

EDITORIAL.

Meeting of the Mississippi Valley Dental Society.... 105
Annual Meeting of the Mississippi Valley Association of Dental Surgeons.... 106
Dental Department of Meharry Medical College.... 107
A New Text Book.... 107

